# Familial invasive and *in situ* squamous cell carcinoma of the skin

**DOI:** 10.1038/sj.bjc.6600909

**Published:** 2003-04-29

**Authors:** K Hemminki, H Zhang, K Czene

**Affiliations:** 1 Department of Biosciences at Novum, Karolinska Institute, 141 57 Huddinge, Sweden; 2 Division of Molecular Genetic Epidemiology, German Cancer Research Center (DKFZ), Heidelberg, Germany

**Keywords:** multiple skin cancer, multiple skin cancers, Bowen's disease, *in situ* carcinoma

## Abstract

We used the updated nation-wide Swedish Family-Cancer Database to examine familial risks in data from 1961 to 1998 on 1252 invasive and 2474 *in situ* squamous cell carcinoma (SCC) of the skin among offspring, and over 10 times more among parents. In 259 families a parent and an offspring had skin SCC. The familial standardised incidence ratios (SIRs) were 2.72 for invasive and 2.40 for *in situ* skin cancers in offspring. Multiple skin cancers in parents were associated with increased SIRs for invasive SCC in offspring, being 2.55 for one and up to 14.93 for two invasive and two *in situ* cancers in parents; the corresponding *in situ* SCC risks were 2.28 and 7.49. The population attributable fraction for any familial skin SCC, invasive or *in situ*, was 4.1%. Melanoma was the only discordant tumour that was associated with invasive and *in situ* skin SCC. These results provide evidence that there is an underlying hereditary susceptibility for at least a part of the familial clustering for skin SCC.

Squamous cell carcinoma (SCC) of the skin is not recorded by most cancer registries and its established risk factors are limited to fair skin, ultraviolet light, arsenic compounds and immunosuppression, while less is known about benign lesions, such as actinic keratosis and *in situ* carcinoma (intraepidermal SCC, or Bowen's disease) (IARC, 1990, IARC, 1995; English *et al*, 1997) The Swedish Cancer Registry has recorded both invasive and *in situ* SCC whose incidence has rapidly increased over the past 20 years (Center for Epidemiology, 2000; Wassberg *et al*, 2001). Skin SCC is the fourth most common cancer in men and women in Sweden in 1998, and the *in situ* form is the most common benign/precancerous tumour in men and the second among women after *in situ* cervical cancer. *In situ* skin cancer does not appear to be a simple precursor lesion to invasive SCC because the age of onset of the two forms is similar; a true precursor lesion would be expected to show an earlier age of onset, as with cervical *in situ* and invasive cancers (MacKie, 1996; Reese, 1998, Reese, 2002; Hemminki *et al*, 1999; Heaphy and Ackerman, 2000; Hemminki and Dong, 2000c; Rees, 2002).

We have previously reported on familial skin cancer from the nation-wide Swedish Family-Cancer Database (Hemminki and Dong, 2000a). In comparisons of familial cancers at many sites in the Database, the standardised incidence ratio (SIR) for invasive skin cancer (2.4) was intermediate, but, it exceeded that for breast (SIR 1.85) and colon (1.89) cancers (Dong and Hemminki, 2001a). We have used the most recent update of the nation-wide Swedish Family-Cancer Database, covering 10.2 million individuals and over 1.0 million tumours to investigate familial relations of invasive and *in situ* SCC. Association of skin SCC with other cancers in families is also described. In contrast to our previous study, the number of cases has almost doubled in the offspring generation, allowing separate analyses for familial risks by invasive and *in situ* SCC, as well as by multiple skin tumours.

## SUBJECTS AND METHODS

The subjects were identified from the Family-Cancer Database, where the first generation (parents) was regarded as probands and familial risks were calculated for the second generation (offspring). We analysed the risk of invasive and *in situ* SCC in offspring by the same or discordant cancer in parents.

### The Swedish Family-Cancer Database

The Swedish Family-Cancer Database, updated in 2000, includes persons born in Sweden after 1931 with their biological parents, totalling over 10.2 million individuals and organised in 3.2 million families (Hemminki *et al*, 1998; Hemminki and Vaittinen, 1998b). Cancers, including *in situ* skin cancers (‘precancerous epithelial lesions’), were retrieved from the nation-wide Swedish Cancer Registry from years 1961 to 1998. The completeness of cancer registration in the 1970s has been estimated to be over 95%, and is now considered to be close to 100%. The percentage of cytologically or histologically verified cases of cancer has been close to 100% (Center for Epidemiology, 2000).

A four-digit diagnostic code based on the seventh revision of the ICD-7 has been used since 1958, together with a code for histological type (WHO/HS/CANC/24.1 Histology Code) in broad subgroups, such as adenocarcinoma and SCC, and we refer to this as the ‘PAD code’, for pathologic anatomic diagnosis. From year 1993 onwards ICD-O-2/ICD with histopathological data according to the Systematised Nomenclature of Medicine (SNOMED, http://snomed.org) was used which we refer to as ‘SNOMED’. Only the first invasive skin cancer or *in situ* cancer was considered, if not stated otherwise. Among discordant cancers in parents, the following ICD-7 codes were pooled: 140 (lip), 141 (tongue), 143–148 (mouth, pharynx) and 161 (larynx), and ‘leukaemia’ 204–207 (leukaemias), 208 (polycytemia vera) and 209 (myelofibrosis). Only adenocarcinoma was included for colorectum and only melanomo for eye cancer.

### Statistical analysis

Family history information was collected on all first-degree relatives (parents, siblings and children) but only the parent–offspring relationship was used here because of the small numbers of affected sibling pairs. There were only one sibling pair with invasive SCC and four pairs with *in situ* SCC and these were included separately in the analysis of familial risk. Standardised incidence ratios, calculated as the ratio of observed (O) to expected (E) number of cases, were used to measure the cancer risks for offspring by occurrence of cancers in their parents. The expected numbers of cancers were obtained by assuming that these persons had the same incidence as in the corresponding general population in the Database: offspring were included if their first primary cancer was diagnised at ages 0–66 years, but no limit was placed on the age of parents. Tumor site-, sex-, 5-year-age- and 10-year-period-specific rates were applied to the relevant person-years at risk (Esteve *et al*, 1994). Person-years at risk were accumulated for each offspring beginning with the date of birth or January 1, 1961 and ending with the date of diagnosis of a first primary cancer, date of death, date of emigration, or December 31, 1998 whichever was earliest. Confidence intervals (95% CI) were calculated assuming that the numbers of cancer cases among offspring follow a Poisson distribution (Esteve *et al*, 1994). The population attributable fraction (PAF) of cases with a family history of invasive or *in situ* skin cancer was estimated as follows: proportion of cases with a family history × (familial SIR−1)/familial SIR, as defined by Miettinen (1974) and cited as formulas (16)–(21) by Rothman and Greenland (1998).

## RESULTS

Table 1

**Table 1:** Numbers of skin cancer by histopathological type

	**Number in offspring**			**Number in parents**		
**Cancer type^a^**	**Son**	**Daughter**	**Offspring**	**Father**	**Mother**	**Parents**
Invasive SSC	728	524	1252	10 945	6109	17 054
Invasive SSC (SNOMED)	513	346	859	4887	3272	8159
						
*In situ* SCC	1248	1226	2474	13 397	13 602	26 999
Mixed type	88	77	165	849	544	1393
Actinic keratosis (SNOMED)	63	72	135	3979	4116	8095
*In situ* SCC (SNOMED)	483	415	898	619	741	1360
Bowen's disease (SNOMED)	257	289	546	1588	1849	3437

a Follow-up for SNOMED, 1993–1998.

presents details from the Family-Cancer Database of 1252 invasive skin SCCs in offspring and 17 054 in parents, together with 2474 and 26 999, respectively of *in situ* SCCs. Age-standardised incidence of invasive and *in situ* SCC is shown in Figure 1

**Figure 1:**
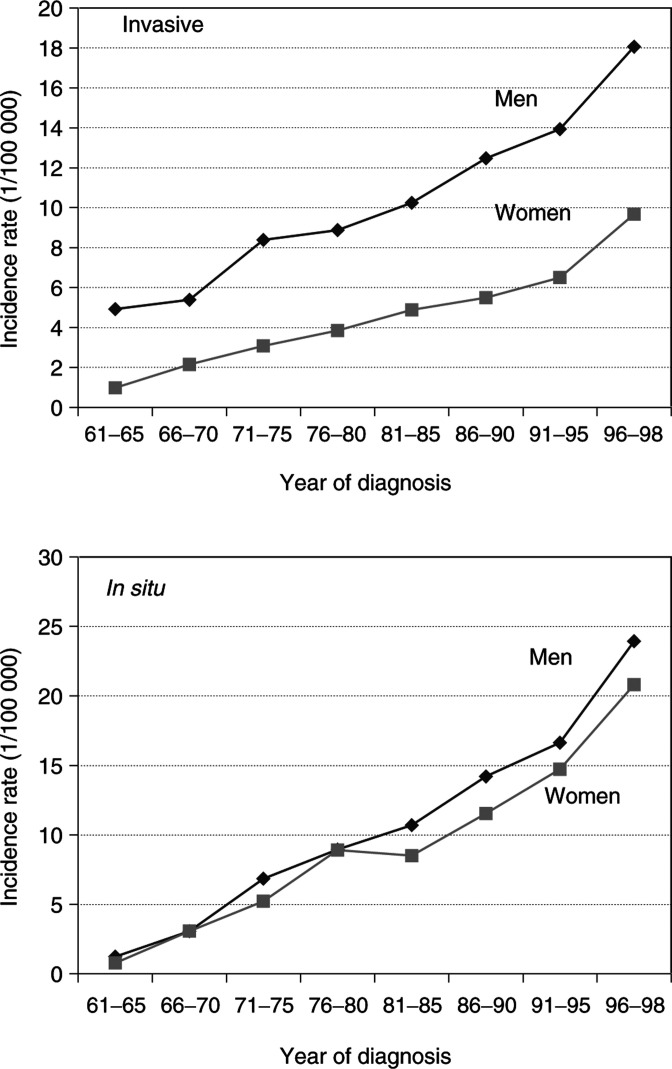
Age-standardised incidence rate of invasive and *in situ* SCC for the period 1961–1998 (standardised according to European standard population)

for men and women. Male rates are almost two times higher for invasive cancer, but for *in situ* cancer there is no difference between the genders. The dramatic increase is seen for all rates, and particularly for *in situ* cancer, the incidence of which exceeds that of invasive SCC at the end of the study period.

Table 2

**Table 2:** Standardised incidence ratio for SCC in offspring of parents with skin cancer

	**SCC in parent**				***In situ* SCC in parent**			
**Skin cancer in offspring^a^**	**O**	**SIR**	**95%**	**CI**	**O**	**SIR**	**95%**	**CI**
Invasive SSC	48	**2.72**	**2.01**	**3.61**	55	**2.24**	**1.69**	**2.92**
Invasive SSC (SNOMED)	35	**3.35**	**2.33**	**4.66**	42	**2.90**	**2.09**	**3.93**
								
*In situ* SCC	83	**2.38**	**1.89**	**2.95**	116	**2.4**	**1.98**	**2.88**
*In situ* SCC (SNOMED)	29	**2.78**	**1.86**	**3.99**	35	**2.44**	**1.70**	**3.39**
Mixed type	2	0.88	0.08	3.25	11	**3.47**	**1.72**	**6.23**
Actinic keratosis (SNOMED)	5	2.51	0.79	5.90	7	**2.60**	**1.03**	**5.38**
Bowen's disease (SNOMED)	16	**2.14**	**1.22**	**3.49**	23	**2.21**	**1.40**	**3.33**

a Follow-up for SNOMED, 1993–1998. Bold type: 95% CI does not include 1.00. O, observed; SIR, standardised incidence rate.

shows SIRs of 2.72 (95% CI 2.01–3.61) for offspring invasive SCC when parents had invasive skin cancer, and 2.24 (1.69–2.92) when they had *in situ* SCC by the PAD code. According to the SNOMED code, the corresponding SIRs were 3.35 (2.33–4.66) and 2.90 (2.09–3.93), respectively. The SIRs for offspring *in situ* SCCs were 2.38 (1.89–2.95) and 2.40 (1.98–2.88) when parents had invasive and *in situ* cancer, respectively. For the mixed type of *in situ* cancer (basal cell and SCC) when parents had *in situ* SCC, the SIR was 3.47 (1.72–6.23), which was the highest SIR found. The SNOMED classification separated three larger groups of *in situ* cancers, actinic keratosis, *in situ* SCC and Bowen's disease with SIRs between 2.14 (1.22–3.49) and 2.78 (1.86–3.99). Among a total of 1252 offspring with invasive skin SCC, 48 (3.8%) had an affected parent and using this proportion and the familial SIR of 2.72, the PAF for familial risk was 2.4%. Similarly, 116 of 2474 (4.7%) offspring with *in situ* tumour had a parent with *in situ* SCC and using 2.40 for the familial SIR, the PAF for *in situ* familial SCC was 2.7%.

When invasive and *in situ* skin SCC were considered together (Table 3

**Table 3:** Standardised incidence ratio for SCC in offspring of parents with skin cancer

**Age group (y)**	**O**	**SIR**	**95%**	**CI**
20–29	5	**3.32**	**1.05**	**7.81**
30–39	15	**1.92**	**1.07**	**3.17**
40–49	71	**2.31**	**1.81**	**2.92**
50–59	131	**2.36**	**1.97**	**2.80**
60–66	37	**2.15**	**1.52**	**2.97**
				
All	259	**2.29**	**2.02**	**2.59**

Bold type: 95% CI does not include 1.00. O, observed; SIR, standardised incidence rate.

), 259 of 3586 (7.2%) offspring with SCC had parents with SCC, resulting in an SIR of 2.29 (95% CI 2.02–2.59) and a PAF of 4.1%. In Table 3, age-specific data for familial skin SCC are also shown. The highest risk was noted at an early age of 20–29 years with SIR of 3.32 (1.05–7.81), while at later ages the SIRs were between 1.92 (1.07–3.17) and 2.36 (1.97–2.80).

Multiple skin cancers in parents, including invasive and *in situ* SCC skin cancers (Table 4

**Table 4:** Standardised incidence ratio for SCC in offspring of parents with skin cancers

	**SCC in offspring**				***In situ* SCC in offspring**			
**Parental SCC**	**O**	**SIR**	**95%**	**CI**	**O**	**SIR**	**95%**	**CI**
1 invasive	42	**2.55**	**1.84**	**3.45**	71	**2.18**	**1.70**	**2.74**
1 *in situ*	46	**2.14**	**1.56**	**2.85**	97	**2.28**	**1.85**	**2.78**
1 invasive and 1 *in situ*	3	1.83	0.34	5.41	9	**2.77**	**1.26**	**5.28**
2 invasive	6	**4.09**	**1.47**	**8.96**	12	**4.10**	**2.11**	**7.19**
2 *in situ*	9	**2.99**	**1.36**	**5.70**	18	**3.02**	**1.78**	**4.78**
2 invasive and 1 *in situ*	4	**7.25**	**1.89**	**18.74**	6	**5.43**	**1.96**	**11.90**
1 invasive and 2 *in situ*	3	4.29	0.81	12.69	6	**4.30**	**1.55**	**9.42**
2 invasive and 2 *in situ*	3	**14.93**	**2.82**	**44.2**	3	**7.49**	**1.41**	**22.18**

Bold type: 95% CI does not include 1.00. O, observed; SIR, standardised incidence rate.

), conveyed an increased SIR to offspring when compared to those of parents with a single skin cancer. The SIR was 14.93 (2.82–44.20) for offspring invasive SCC when a parent had two invasive and two *in situ* cancers, the comparable *in situ* SCC risk was 7.49 (1.41–22.18). However, the number of affected offspring was small when parents had multiple skin cancers.

Familial risks of SCC in offspring were analysed when parents had diverse invasive cancers (Table 5

**Table 5:** Standardised incidence ratio for SCC in offspring by type of parental cancer

	**SCC in offspring**				***In situ* SCC in offspring**			
**Parental invasive**	**O**	**SIR**	**95%**	**CI**	**O**	**SIR**	**95%**	**CI**
Upper aerodiagestive tract	16	1.13	0.64	1.84	21	0.70	0.44	1.08
Oesophagus	7	1.38	0.55	2.87	6	0.56	0.20	1.23
Stomach	28	0.77	0.51	1.11	66	0.85	0.66	1.08
Colon	77	1.13	0.89	1.41	118	0.82	0.68	0.99
Liver	14	0.66	0.36	1.12	26	0.58	0.38	0.86
Pancreas	23	1.11	0.70	1.67	34	0.78	0.54	1.09
Lung	46	1.11	0.81	1.48	85	0.99	0.79	1.22
Breast	60	0.99	0.76	1.28	110	0.88	0.72	1.06
Cervix	18	1.48	0.88	2.35	15	0.60	0.34	0.99
Endometrium	17	1.13	0.66	1.81	21	0.67	0.42	1.03
Ovary	10	0.70	0.33	1.30	26	0.88	0.58	1.30
Prostate	106	**1.24**	**1.01**	**1.50**	180	1.00	0.86	1.16
Kidney	19	0.92	0.55	1.44	37	0.86	0.60	1.19
Urinary bladder	42	**1.49**	**1.08**	**2.02**	57	0.97	0.74	1.26
Melanoma	19	**1.78**	**1.07**	**2.79**	33	**1.53**	**1.06**	**2.16**
Skin SCC	48	**2.72**	**2.01**	**3.61**	83	**2.38**	**1.89**	**2.95**
Eye melanoma	5	**3.26**	**1.03**	**7.68**	4	1.25	0.32	3.22
Nervous system	9	0.60	0.27	1.14	35	1.13	0.79	1.57
Thyroid gland	6	1.39	0.50	3.04	7	0.78	0.31	1.61
Endocrine glands	10	1.14	0.54	2.10	25	1.40	0.90	2.06
Non-Hodgkin's lymphoma	16	1.02	0.58	1.67	31	0.96	0.65	1.36
Myeloma	5	0.50	0.16	1.19	27	1.29	0.85	1.88
Leukaemia	15	0.88	0.49	1.45	40	1.11	0.79	1.52
								
Total	616	**1.13**	**1.04**	**1.22**	1087	0.96	0.90	1.02

a Cancer sites with less than five cases are excluded. Bold type: The lower 95% CI does not include 1.00. O, observed; SIR, standardised incidence rate.

). Among the parental sites, the urinary bladder, prostate, skin melanoma, skin SCC and eye melanoma were associated with invasive SCC in offspring. For offspring *in situ* SCC, only parental melanoma and invasive SCC increased the risk. The data in Table 5 were derived from 46 comparisons among cancer sites, which could have resulted in 2 or 3 significant findings owing to chance alone. We found nine significant differences, of which seven were increases, suggesting that some increases were genuine.

## DISCUSSION

The only previous study of familial skin SCC is that by Hemminki and Dong (2000a). Our present study is substantially larger, allowing analysis of both offspring and parents by the squamous cell histology. Some possible biases need to be considered. The quality of the Swedish Cancer Registry is high, but the marked increas particularly of *in situ* cases (Figure 1) raises the possibility that reporting to the Registry has improved over the time. All registered cases of SCC were verified by histology or cytology (Center for Epidemiology, 2000). At present there may be an increased concern and alertness in families with affected individuals and may be seeking medical opinion about treatment more often than in the past. This cannot be excluded in Sweden for SCC but as the diagnoses are always confirmed by histology or cytology, the effect would be a lead-time bias rather than a false diagnosis (Hemminki and Vaittinen, 1998a; Wassberg *et al*, 1999). The numbers of affected types of families argue against bias: as compared to 259 affected parent–offspring families with any skin SCC, there was only one sibling pair with invasive SCC and four pairs with *in situ* SCC. Considering the number of skin tumours in the 3.2 million families of the Database, the number of affected sibling pairs was within expectations.

The data on family clustering of skin SCC show a strong effect with a SIR of 3.35 for concordant invasive SCC in parents and offspring, based on the SNOMED codes used during 1993–1998 although the corresponding risk for the whole period, 1961–1998, was somewhat lower at 2.72. These risks, together with the very high SIRs from multiple tumours in the parental probands, such as 7.25 from three tumours and 14.93 from four tumours provide strong evidence for heritable effects in skin SCC. The familial SIRs for *in situ* SCC were also increased but tended to be lower than those for invasive tumours, which could imply a degree of diagnostic inconsistency. Both invasive and *in situ* form were represented in the same families giving further evidence of their biological similarity. The mixed *in situ* tumour with basal and squamous cell elements showed the highest SIR of all comparisons, 3.47 from parental *in situ* SCC. The three types of *in situ* tumours, in the SNOMED classification were comparable in their familial presentation, as far as the number of cases allowed any conclusions. In the dermatological literature there has been a long standing controversy as to the distinction of the different kinds of *in situ* tumours and whether they represent different biological entities between each other and between them and invasive forms (MacKie, 1996; Heaphy and Ackerman, 2000; Rees, 2002).

When invasive and *in situ* skin cancer were considered together, familial risk was higher at young ages and the PAF of familial SCC was 4.1%. Familial clustering may be because of inherited and/or environmental factors. One way to assess the shared environmental factors involving exposure to sun is to compare the correlation of skin SCC between spouses and the fact that no such correlation has been found in this Database and this comparison suggests that heritable factors are more important than environmental factors in the familial aggregation of skin cancer (Hemminki *et al*, 2001a; Hemminki and Jiang, 2002). Moreover, in a separate study we have found that familial risk can be found both in sun-exposed and covered sites, adding to the evidence that sun exposure alone does not explain the familial aggregation (unpublished data). The molecular basis of SCC is not fully understood (Reese, 1998, Reese, 2002; Rees, 2002), mutations in the *PTC* gene, common in basal cell carcinoma, being rare in SCC. Although *p53* and H-*ras* mutations are common they may not be the initial events in skin carcinogenesis. Squamous cell carcinoma is not a feature of Li-Fraumeni families who have a germ-line mutation in the *p53* gene (Malkin, 1998). Since sun exposure is an established risk factor of skin cancer, at least part of the familial risk may be explained by cumulative exposure to sun, or by an inherited trait of skin type. SCC is a major complication in two rare recessive syndromes: xeroderma pigmentosum and Bloom syndrome, involving defects in DNA excision repair and DNA helicase, respectively (German and Ellis, 1998; Balajee and Bohr, 2000; de Boer and Hoeijmakers, 2000). Since these syndromes are recessive, they would not be observed when comparing familial cancers between parents and offspring. The important question of a possible cancer risk in the heterozygous carriers in the DNA repair gene defects awaits further study.

Melanoma was the only discordant tumour that associated with invasive and *in situ* skin SCC, an association also noted in melanoma families (Hemminki *et al*, 2001b). In the present study, the excess familial risks for invasive and *in situ* SCC in offspring were 2.2 and 2.6 times higher, respectively, when a parent had the same cancer as compared to a parent with melanoma. Solar irradiation affects SCC and melanoma in different ways and the distribution of affected body parts also varies (English *et al*, 1997). However, considering the much higher risks from parental SCC than melanoma, some contribution by ultraviolet radiation to the SCC–melanoma association is likely. Even more interesting was the association of invasive SCC (SIR 3.26) with eye melanoma, constituting some 80% of intraocular tumours, for which sun ultraviolet radiation has been considered only a weak risk factor (English *et al*, 1997). Sweden is located between latitudes 55 and 70°, implying that the prevailing low solar altitude causes a relatively long-lasting ultraviolet radiation exposure of the retina. A further point is that eye melanoma does not appear to associate with familial cutaneous melanoma (Sinilnikova *et al*, 1999; Hemminki and Jiang, 2001). These data suggest that solar irradiation is the common denominator for the aggregation of skin SCC, cutaneous melanoma and ocular melanoma, and this is supported by recent reports (Shors *et al*, 2002; Vajdic *et al*, 2002; Hemminki *et al*, 2003). The significance of other associations between parental prostate and bladder cancers with invasive SCC remains unclear.

In contrast to the scarcity of familial data on skin SCC, there are many studies published on second cancers following skin SCC (Frisch and Melbye, 1995; Levi *et al*, 1996, Levi *et al*, 1997, Levi *et al*, 1998; Wassberg *et al*, 1999; Dong and Hemminki, 2001b; Hemminki and Dong, 2000b, Hemminki and Dong, 2000c; Milan *et al*, 2000). These have shown a consistent link between SCC and melanoma, while an association between skin and eye cancer has been noted (Milan *et al*, 2000). Many studies on second cancers have found an excess of cancers associated with immunological disturbances and immunosuppression, including lymphoma, and lip (oral), cervical and connective tissue cancers (IARC, 1996). However, the present results provide no support for the role of impaired immune function.

In summary, the present data on relatively young individuals provided evidence that there is an underlying hereditary susceptibility explaining at least a part of the familial clustering of skin SCC. Familial risks between invasive and various *in situ* forms of SCC appear to be similar and in many families different forms of SCC are manifested. The association of skin SCC with cutaneous and ocular melanoma may be largely because solar irradiation.
